# Impact of Different Mucoadhesive Polymeric Nanoparticles Loaded in Thermosensitive Hydrogels on Transcorneal Administration of 5-Fluorouracil

**DOI:** 10.3390/pharmaceutics11120623

**Published:** 2019-11-21

**Authors:** Angela Fabiano, Anna Maria Piras, Lorenzo Guazzelli, Barbara Storti, Ranieri Bizzarri, Ylenia Zambito

**Affiliations:** 1 Department of Pharmacy, University of Pisa Via Bonanno, 33, 56126 Pisa, Italy; anna.piras@unipi.it (A.M.P.); lorenzo.guazzelli@unipi.it (L.G.); 2 NEST, Scuola Normale Superiore and Istituto Nanoscienze-CNR, Piazza San Silvestro 12, 56127 Pisa, Italy; barbara.storti@nano.cnr.it (B.S.); ranieri.bizzarri@nano.cnr.it (R.B.); 3 Department of Surgical, Medical and Molecular Pathology, and Critical Care Medicine, University of Pisa, Via Roma 67, 56126 Pisa, Italy

**Keywords:** thermosensitive hydrogels, mucoadhesive chitosan multifunctional derivatives, mucoadhesive nanoparticles, ocular cancer, microrheology, ocular delivery

## Abstract

In a previous paper a thermosensitive hydrogel formulation based on chitosan or its derivatives (TSOH), containing medicated chitosan nanoparticles (Ch NP) for transcorneal administration of 5-fluorouracil (5-FU) was described. The Ch NP-containing TSOH allowed a time-constant 5-FU concentration in the aqueous for 7 h from instillation. The aim of the present work was to study the impact of the surface characteristics of new NP contained in TSOH on ocular 5-FU bioavailability. The Ch derivatives used to prepare NP were quaternary ammonium-Ch conjugate (QA-Ch), *S*-protected derivative thereof (QA-Ch-*S*-pro), and a sulphobutyl chitosan derivative (SB-Ch). All NP types had 300–400 nm size, 16–18% encapsulation efficiency, and retained the entrapped drug for at least 15 h. Drug release from TSOH containing NP based on QA-Ch or QA-Ch-*S*-pro was virtually equal, whereas with TSOH containing NP based on SB-Ch was significantly slower. Instillation, in rabbit eyes, of NP-containing TSOH based on QA-Ch or SB-Ch led to a plateau in the aqueous concentration vs. time plot in the 1–10 h range with significantly enhanced area under curve (AUC). Negative charges on the NP surface slowed down 5-FU release from TSOH while positive charges increased NP contact with the negatively charged ocular surface. Either results in enhanced ocular bioavailability.

## 1. Introduction

Topical ophthalmic preparations such as eyedrops are the most commonly used ocular drug delivery systems. Unfortunately, the intraocular bioavailability of their active substances is generally less than 5%, due to the anatomical and physiological characteristics of the eyes, including tissue barriers, such as cornea, lens, conjunctiva, and sclera, and various physiological functions, such as lacrimation and consequent dilution, and drug expulsion by tear turnover. As a consequence, to obtain the appropriate intraocular drug concentrations, frequent instillations of eyedrops are needed, which can cause toxic side effects and damage to the ocular tissue [1]. Thus, the traditional ophthalmic preparations and relevant therapeutic protocols cannot provide and maintain effective drug concentrations in the corneal tissue, and this results in a poor ocular bioavailability.

The intraocular bioavailability would improve if the drug transcorneal permeability and precorneal residence were increased [2]. One strategy to improve the corneal permeability is the addition of polymeric penetration enhancers to eyedrop formulations [3,4,5].

Another interesting approach to ocular drug delivery is the use of hydrogel systems. Among hydrogels the most interesting for the ocular administration of drugs are the thermosensitive hydrogels that can be instilled in the form of drops and become gels once in contact with the eyes [6,7,8]. In particular, chitosan-based thermosensitive hydrogels have proved to be vehicles of choice for biomedical application because they possess mucoadhesive, antibacterial, biodegradable, and biocompatible properties [7,9].

Another approach aimed at increasing the endo-ocular bioavailability of drugs is the use of nanoparticles (NP) or mucoadhesive vesicles capable of prolonging the contact with eye mucosae and promoting the absorption of entrapped drugs [10,11]. Furthermore, the nanoparticles could be internalized by the corneal cells, and form a reservoir from which the drug could be released over time [12].

Recently, innovative formulations based on thermosensitive hydrogels for the sustained ocular delivery of drugs entrapped in nanosize structures have been proposed [13,14,15]. In all cases such systems were found to have a greater ability than those based on the simple thermosensitive hydrogels to prolong the drug residence in the precorneal area, promote endoocular bioavailability, and prolong the drug halflife in the aqueous. If nanosystem diffusion in hydrogels is excluded, the above results can be explained by the blinking continuously renewing the hydrogel-cornea contact surface, thus bringing new NP into contact with the cornea.

In our previous work we tried to combine the above strategies by preparing thermosensitive hydrogels (TSOH) based on chitosan and its derivatives containing medicated NP based on unmodified chitosan for transcorneal administration of 5-fluorouracil (5-FU) [13]. Chitosan was chosen because cationic polymers have greater interactivity with the negatively charged ocular surface [16], and also, because chitosan has a lower toxicity than other cationic polymers, such as polyarginine or polyethylenimine [17]. Increased bioavailability of 5-FU in the aqueous with respect to control eyedrops resulted from in vivo experiments with rabbit eyes. The NP-containing hydrogels mediated to a zero-order 5-FU absorption, leading to a time-constant anticancer concentration in the aqueous for up to 7 h from instillation. This was ascribed to the ability of this TSOH to control drug release to a zero order and that of NP to be internalized by corneal cells. In that first study the preparation of thermosensitive hydrogels was studied and developed, while the impact of the surface characteristics of the NP they contained on drug ocular bioavailability was not studied. Such an impact has been investigated in the present study.

Nanoparticles differing in mucoadhesivity and surface charge were prepared for this purpose from chitosan (Ch) derivatives, and NP ability to promote the transcorneal penetration of 5-FU was assessed in vivo. The chitosan derivatives consisted in quaternary ammonium-Ch conjugate (QA-Ch), *S*-protected derivative thereof (QA-Ch-*S*-pro), and a sulfobutyl chitosan derivative (SB-Ch). While the properties of quaternary ammonium-Ch conjugate and/or those of the respective *S*-protected derivatives have widely been described in the literature, the properties of Ch modified by sulfate or sulfonate groups is currently under investigation [18]. The NP were dispersed in TSOH in the sol state and compared on the ground of NP size, surface zeta potential, mucoadhesivity, affinity with the drug, and in vitro drug-release properties, in order to select the systems on which to carry out in vivo pharmacokinetic studies.

## 2. Materials and Methods

### 2.1. Materials

Low molecular weight Ch (75–85% deacetylated), 5-FU, β-glycerophosphate (β-GP) disodium salt, sodium tripolyphosphate (TPP), 1,4-butane sulphone, and fluorescein isothiocyanate (FITC), Type II mucin from porcine stomach, were purchased from Sigma-Aldrich (Milan, Italy). The QA-Ch conjugate was synthesized from Ch according to Zambito et al., 2013 [19], the thiolated *S*-protected derivative of QA-Ch was synthesized according to Fabiano et al., 2018 [20]. Reduced molecular weight hyaluronic acid (rHA) (viscosimetric molecular weight 470 kDa) was prepared as described by Zambito et al., 2013 [19]. FITC labelling of QA-Ch, QA-Ch-*S*-pro, and SB-Ch was carried out as previously described [21]. The QA-Ch50 polymer, used to prepare the TSOH, was synthesized according to Zambito et al., 2013 [19]. In the code, 50 means that it was prepared by maintaining the temperature at 50 °C for the entire duration of the reaction. All aqueous solutions/dispersions were prepared with freshly distilled water. QA-Ch was characterized by ^1^H NMR to determine the degree of substitution with the small side chains containing adjacent quaternary ammonium groups and the length of such chains. Protected thiols present on QA-Ch-*S*-pro chains were determined by polymer reduction and subsequent quantification of the 6-mercaptonicotinamide protecting group. Only polymers that had the same characteristics as previously obtained [20] were used in this work.

### 2.2. Synthesis and Characterization of Sulphobutyl Chitosan (SB-Ch)

**Figure 1:**
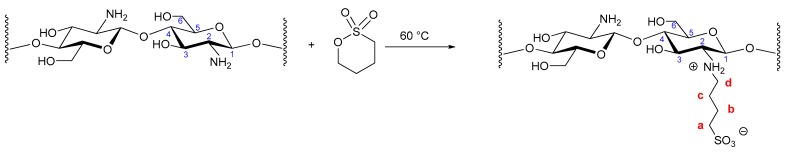
Synthetic route to sulfobutyl chitosan (SB-Ch).

SB-Ch was synthesized from Ch as previously described [22]. Briefly, 1,4-butane sulphone (3 equivalents per *N*-acetylglucosamine unit) was added to a Ch solution in acidic water (1% *w*/*w* Ch, 2% *w*/*w* of acid acetic). The mixture was allowed to react at 60 °C for 6 h. The resulting solution was poured into acetone. The precipitated product was resuspended in demineralized water and purified by dialysis 3 days against water. After dialysis, the polymer solution was lyophilized to obtain the purified SB-Ch (Figure 1). ^1^H NMR spectrum was recorded in D_2_O/DCl with a Bruker AC 200 instrument operating at 200.13 MHz (Bruker, Milan, Italy).

### 2.3. Preparation of Medicated NP

FITC-labeled or unlabeled NP based on QA-Ch or QA-Ch-*S*-pro were prepared by self-assembly upon addition of rHA. In detail, a solution of rHA 0.2 mg/mL in phosphate buffer (0.13 M, PB pH 7.4) containing 6.25 mg of 5-FU was added dropwise (500 μL) to 5 mL of 2 mg/mL polymer solution in the same buffer, under stirring at room temperature. Similarly, to prepare FITC-labeled or unlabeled NP based on SB-Ch, a solution of TPP 2 mg/mL in demineralized water containing 6.25 mg of FU was added dropwise (700 μL) to 5 mL of 2 mg/mL SB-Ch in demineralized water, under stirring at room temperature. The final 5-FU concentration in NP systems based on QA-Ch, or QA-Ch-*S*-pro, or SB-Ch was 1.25 mg/mL, corresponding to the concentration contained in the commercial 5-FU eyedrops. After their preparation, the NP dispersions were checked for particle size and zeta potential (ζ) at a temperature of 25 °C (Zetasizer Nano ZS, Malvern, UK). The ζ values of medicated NP based on QA-Ch, QA-Ch-*S*-pro, or SB-Ch were determined after NP centrifugation (2000 rpm for 30 min) and their subsequent sediments re-suspended in 1.9 mL of a 0.08 M HCl solution containing 195 μL of NaOH 1 N and 0.8 g/mL of β-GP. Their drug-entrapment efficiency (EE) was evaluated by subjecting the dispersion to centrifugation (20,000 rpm for 30 min at 4 °C) and analyzing the supernatant spectophotometrically at 266 nm. The EE was calculated as follows, using the appropriate calibration curve:

EE = (M_t_ − M_s_)/M_t_ (1)

where M_t_ is the total mass of 5-FU used for the preparation of NP and M_s_ is the mass found in the supernatant.

### 2.4. Preparation of Thermosensitive Hydrogels (TSOH) Containing NP Medicated with 5-FU

Thermosensitive ophthalmic hydrogels (TSOH) were prepared according to Fabiano et al. [13]. Briefly, 400 mg of Ch and 100 mg of QA-Ch50 were dissolved in 18 mL of a 0.08 M HCl solution. The resulting solution was kept under magnetic stirring at 4 °C. Then, 5-FU-medicated NP, based on QA-Ch, QA-Ch-*S*-pro, or SB-Ch, freshly prepared, were added in the sol state under magnetic stirring at 4 °C, before the addition of 450 µl β-GP (0.8 g/mL) solution to obtain TSOH.

### 2.5. Dynamic Dialysis Studies

To study the reversible drug binding by NP in fluid dispersion we used already reported procedure and theory [23,24]. Briefly, a porous cellulose membrane (cut-off 12.5 kDa) was used to separate the donor compartment of the dialysis cell from the receiving phase (100 mL of PB pH 7.4 for 5-FU-loaded QA-Ch or QA-Ch-*S*-pro NP or100 mL of demineralized water for 5-FU-loaded SB-Ch NP). The system was thermostated at 35 °C for 5 h, while maintaining sink conditions. At time *t* = 0, 5 mL of freshly prepared 5-FU loaded NP, or plain drug solution (control), or this solution containing 2 mg/mL of QA-Ch or QA-Ch-*S*-pro dissolved in PB pH 7.4, or 2 mg/mL of SB-Ch in water was introduced in the donor compartment of the cell. In all cases, at the end of experiment, the receiving phase was analyzed spectrophotometrically to determine the drug transport. In the case of NP, the receiving phase was also analyzed for particle size.

### 2.6. Interrupted-Dialysis Studies

The dynamic dialysis experiment, described in Section 2.5, was stopped after 1, 2, 3, 5, 15, or 24 h, from the start. At each time interval, the donor phase was centrifuged (2000 rpm for 30 min at 4 °C) to determine the drug fraction contained in NP matrix, NP dispersion medium, and acceptor medium, according to reference [25]. The results were plotted as drug fraction in each phase versus time.

### 2.7. Studies of 5-FU Release from NP-Containing TSOH

5-FU release from TSOH was carried out using a cell and a procedure reported by Fabiano et al. [13]. The gel (0.5 mL) containing medicated NP was introduced in the cylindrical cavity of the cell. A porous cellulose membrane (cut-off 12.5 kDa) was used to separate the gel from the receiving phase (30 mL of PB pH 7.4 for 5-FU-loaded NP based on QA-Ch or QA-Ch-*S*-pro, or 30 mL of demineralized water for 5-FU-loaded NP based on SB-Ch). At time, *t* = 0, the cell was introduced in a beaker containing the receiving phase thermostated at 35 °C and stirred at 300 rpm. The receiving phase was analyzed spectrophotometrically at 30-min intervals to determine the drug transport kinetics.

### 2.8. Confocal Microscopy and Image Analysis

To evaluate the possibility of re-dispersing NP into the hydrogel system, the FITC-labelled, 5-FU medicated NP, based on QA-Ch, QA-Ch-*S*-pro, or SB-Ch were dispersed in the TSOH at room temperature and observed under a confocal laser-scanning microscope (Zeiss LSM 880 with Airyscan, Carl Zeiss, Jena, Germany). The representative fluorescence confocal micrographs of NP were taken in liquid (21 °C) and gel states (4 °C), using a 63x Apochromat NA = 1.4 oil-immersion objective with the pinhole aperture of the confocal system at 1 Airy unit. The excitation wavelength was set at 488 nm (10–20 µW power emission at objective), whereas emission was in the 500–550 nm range. Pixel dwell time was adjusted to 1.52 µs and 512 × 512 pixel images were collected.

Image analysis was carried out by ImageJ v.1.52o (NIH, Bethesda, MD, USA) software. Particle diameters were calculated by tracing an equatorial line over each bead (average of 5–10 beads), collecting the fluorescence profile and fitting it with a Gaussian function. The full width at half maximum (FWHM) of the best-fitting curve was assumed as particle diameter.

### 2.9. Micro-Rheological Characterization of NP Mucoadhesive Properties

Micro-rheological measurements were carried out using a Zetasizer Nano ZS, Malvern, with a detection angle of 173 °C and a temperature of 25 °C, applying the theory reported by Dodero et al. [26]. The micro-rheological characterization of 5-FU loaded freshly prepared QA-Ch or SB-Ch NP was performed using mucin from porcine stomach, Type II. Ocular mucins are not commercially available, so porcine gastric mucin was used because it has also been applied as a model substance in other studies, investigating ocular mucoadhesion [27]. In order to obtain reliable micro-rheological data, the conditions about the tracer-sample combination were verified as reported by Dodero et al., 2019 [26]. A dispersion of mucin 3 mg/mL in water, was filtered using a cellulose acetate filter (pore size 0.45 µm). A sample was taken from the filtrate and lyophilized to calculate the concentration of dispersed material (1.85 mg/mL). The filtered mucin dispersion was diluted 10 times with a solution of NaCl 0.9%. Then, 5 µL of tracer sample (polystyrene latex particles, diameter 500 nm, Beckman, 5 µl/mL) and 5 µL of NP dispersions prepared as described in Section 2.4 were added to the diluted mucin dispersion. Micro-rheological tests were performed to evaluate viscoelastic properties and assess NP mucoadhesive properties on the basis of the viscosity changes caused by NP addition to a mucin solution.

### 2.10. In Vivo Studies

For the in vivo studies we used male New Zealand albino rabbits weighing 3–3.5 kg treated as prescribed in the guidelines from the European Community Council Directive 2010/63, approved by the Animal Care Committee of the University of Pisa (D.L. 2014/26, 12 March 2019). Fifty µL (one drop) of the following two types of ophthalmic formulations were instilled in the lower conjunctival sac: (1) a dispersion of QA-Ch-based NP medicated with 1.25 mg/mL of 5-FU in TSOH and (2) a dispersion of SB-Ch-based NP medicated with 1.25 mg/mL 5-FU in TSOH. For the entire duration of the experiments each rabbit eye was checked for signs of conjunctival/cornea edema and/or hyperemia [28]. Before the aspiration of aqueous humor (~60 µL) from the anterior chamber of the eye, the rabbits were anesthetized with one drop of Novesina^®^. The 5-FU concentration in aqueous humor was determined by high-performance liquid chromatography (HPLC) using the apparatus and the mobile phase described by Fabiano et al. [13]. An Aeris 3.6 µm, PEPTIDE XB-C18 Å, 250 × 4.6 mm column, equilibrated at 30 °C was used and UV detection was set at 266 nm. Standard curves were obtained analyzing six standard drug solutions (concentration range 0.3–1.25 µg/mL) in acetonitrile mixed with aqueous humor (2:1). The resulting mixtures were centrifuged, and the acetonitrile was removed by evaporation at 50 °C. The resulting aqueous product was lyophilized and re-dispersed in a volume of mobile phase corresponding to the initial volume of standard solution. Standard curves were linear (*r*^2^ > 0.995, limit of detection 0.2 µg/mL). The retention time was 8.2 min. The concentration of each unknown sample was determined as described above, using a standard curve produced on the same day.

### 2.11. Data Treatment

Linear plots were obtained by linear regression analysis of data from in vitro experiments. The relevant slope, intercept, and coefficient of determination (*r*^2^) were calculated. The significance of differences was evaluated by Student’s t-test (*p* < 0.05). For the in vivo experiments, the linear trapezoidal rule between 0 and 10 h was used to calculate the area under curve (AUC) and the statistical differences were evaluated using the method reported by Schoenwald et al. [29].

## 3. Results and Discussion

### 3.1. Synthesis of Sulphobutyl Chitosan (SB-Ch)

**Figure 2:**
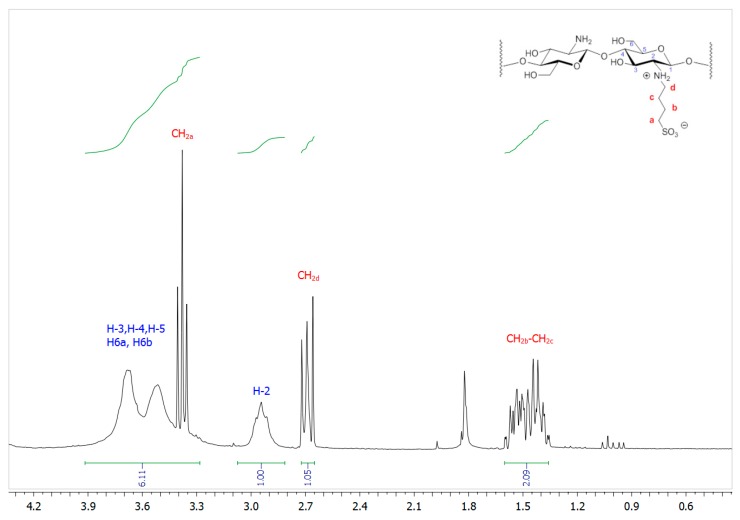
^1^H NMR (200.12 MHz, D_2_O/DCl) spectral region of SB-Ch.

^1^H NMR spectrum (D_2_O/DCl) of SB-Ch, seen in Figure 2, shows three sets of signals ascribed to the different methylene groups of the sulfobutyl functionalization at 1.32–1.60 ppm (–CH_2b_ and –CH_2c_), 2.62–3.72 ppm (–CH_2d_), and 3.33–3.41 ppm (–CH_2a_ overlapping to protons H-3, H-4, H-5, and H-6 of chitosan). An estimation of the butylsulfonation degree (ca 52%) was possible by comparing the integrals of H-2 of the chitosan backbone, which fell in a quite clean part of the spectrum (2.91–3.20 ppm), and of –CH_2d_ of the sulfobutyl group.

### 3.2. Characteristics of Medicated NP

The size, polydispersity index, zeta potential (ζ) and encapsulation efficiency (EE) for medicated NP based on QA-Ch, QA-Ch-*S*-pro, or SB-Ch are found in Table 1. The size and polydispersity index values were not significantly different from each other or from those for NP based on Ch prepared in our preceding paper Fabiano et al. [13] (342.5 ± 15.2). The ζ values for medicated NP based on QA-Ch or QA-Ch-*S*-pro were positive in agreement with the presence of ammonium quaternary ions on their surface, whereas for medicated NP based on SB-Ch the ζ value was negative in agreement with the presence of superficial sulfonic groups. The EE was in the range 18–15% with no significant difference between the three cases. The prepared NP only differing for their external charge allowed us to evaluate the impact of this property on the system ability to promote the intraocular absorption of 5-FU.

### 3.3. Dynamic Dialysis Studies

**Figure 3:**
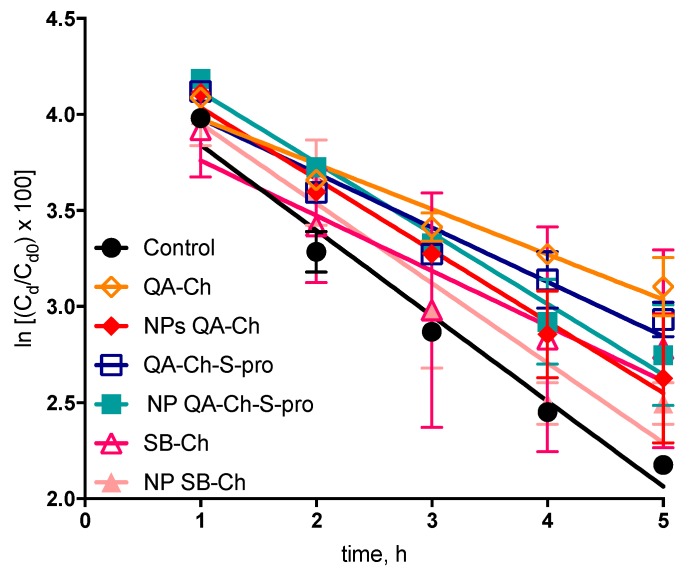
Dynamic dialysis data plotted according to Equation (2). Means ± SD (*n* = 3–6).

Dynamic dialysis data were used to compare reversible drug interactions with the NP in fluid dispersions. According to [23,24], the data obtained were plotted as ln (C_d_/C_d0_) × 100 versus *t* using the following Equation (2):

ln [(C_d_/C_d0_) × 100] = 4.605 − K_m_ F_f_*t* (2)

where C_d_ is the drug concentration in the donor phase, C_d0_ is the drug concentration in donor phase at *t* = 0, F_f_ is the non-interacting drug fraction in donor phase, and K_m_ is the dialysis-rate constant. All plots, reported in Figure 3 were significantly linear (*r*^2^ values, 0.90–0.98) which indicated that in all cases (plain 5-FU, control, NP based on QA-Ch, QA-Ch-*S*-pro, or SB-Ch) Equation (2) was obeyed and for plain 5-FU (control) the slope of the relative log-linear plot, used to calculate F_f_ was equal to K_m_. The slopes of the straight lines reported in Table 2 indicated a 5-FU binding with all polymers in solution higher than the binding with NP dispersions. From here, it was deduced that the drug fraction reversibly interacting with the polymers in solution was significantly higher than that adsorbed on NP surface. This was probably due to an interaction between the 5-FU and the polycationic polysaccharide Ch. In the case of NP, the positive groups of Ch could be bound to the crosslinker and, hence, less available for 5-FU binding. This, nevertheless, should not impair the NP effectiveness in vivo, in fact, nanoparticles made of mucoahesive polymers are themselves more mucoahesive than the corresponding parent polymers, hence, likely to strongly adhere to the ocular surface [10,20,30].

### 3.4. 5-FU Release from NP

**Figure 4:**
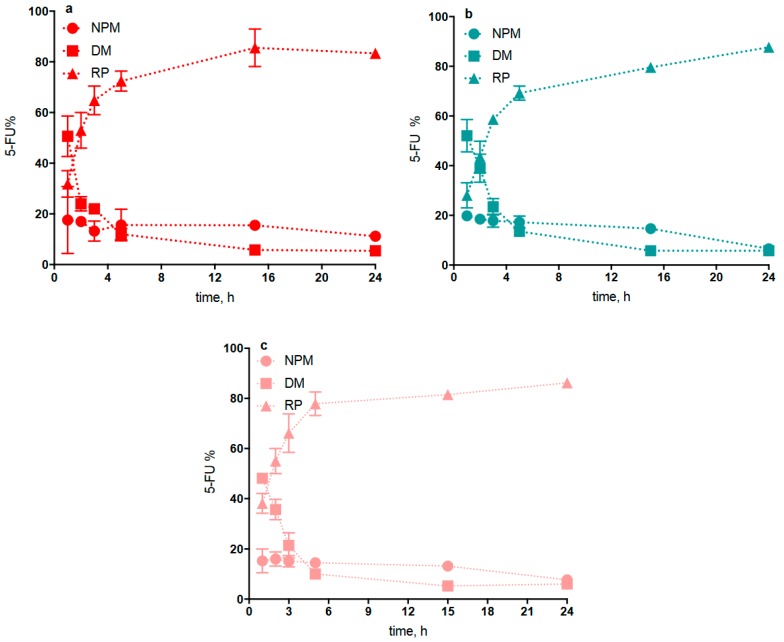
Interrupted dialysis studies: kinetic analysis of dialysis phases for 5-fluorouracil (5-FU)-loaded QA-Ch NP (**a**), QA-Ch-*S*-pro NP (**b**), and SB-Ch NP (**c**). Percent 5-FU in: NP matrix (NPM); NP dispersion medium (DM); and receiving phase (RP). Means ± SD (*n* = 3).

The procedure used to study 5-FU release from NP was based on interrupted dialysis, as described in Section 2.6. A dispersion of each freshly prepared NP type, loaded with 5-FU and not separated from the non-entrapped drug was introduced in the donor compartment of the dialysis cell. Hence, not more than 15–18% of the whole 5-FU amount contained in each dispersion was associated with the NP phase, as shown in Table 1. From knowledge of the drug amount used for each NP preparation, the drug amount determined for the NP dispersion medium in donor compartment (DM phase) at each interruption time, the cumulative amount determined for the drug transferred into the receiving medium (RM phase) during each interruption time, and the relevant % 5-FU contained in the NP matrix, i.e., the NPM data reported in Figure 4, could readily be calculated. This data indicated that the 5-FU fraction immobilized in the NP matrix remained virtually constant at the initial value of 15–18% over the first 15 h of experiment, while it seemed to fade after 24 h probably due to some degradation of the NP. Therefore, it is understood that all NP types are able to retain the 5-FU load for a term sufficient for NP to be internalized by corneal cells. It should be noted that the present NP, prepared from Ch derivatives, were able to retain 5-FU for longer than those prepared by Fabiano et al., from Ch (15 vs. 5 h) [13].

### 3.5. Drug Release from NP-Containing TSOH

**Figure 5:**
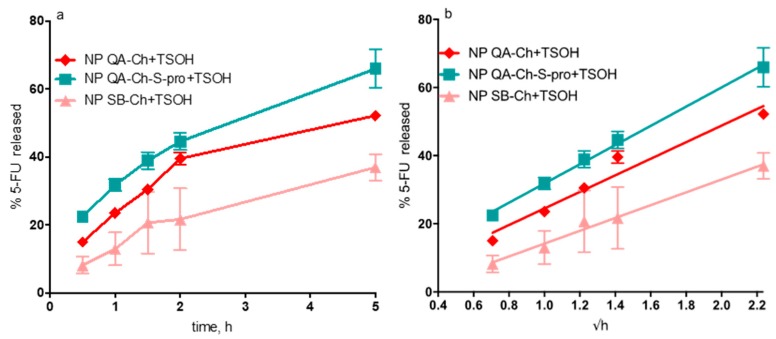
5-FU released (%) from NP-containing thermosensitive hydrogels (TSOH) vs. time (hours) (**a**) or vs. √t (**b**).

Data on drug release from NP-containing TSOH, obtained as described in Section 2.7 and plotted as percentage of drug released versus t or √t are reported in Figure 5a,b, respectively. The release study lasted 5 h since the 5-FU release study for all NP types had shown that the 5-FU % entrapped in the NP was virtually constant for at least 15 h. The drug amount released vs. √t was in all cases linear with comparatively small ordinate intercepts (between −0.99% and 2.88%) and high *r*^2^ values (between 0.96 and 0.99, *n* = 3). This pattern in all cases fitted a well-known model assuming that the release was entirely governed by drug diffusion in the releasing vehicle. The slope of each straight line allowed comparison between the different cases on the basis of drug diffusivity in the hydrogel. The data listed in Table 3 shows that 5-FU release from TSOH containing NP based on QA-Ch or QA-Ch-*S*-pro was not significantly different from drug release from TSOH containing NP based on Ch, whereas drug release from TSOH containing NP based on SB-Ch was significantly slowed down, with respect to the above, reasonably by the negative charges on the NP surface dispersed in this TSOH. Indeed, TSOH was prepared using a chitosan derivative containing fixed positive charges that could electrostatically interact with the negative charges of SB-Ch, and this interaction could slow down the 5-FU release from TSOH.

### 3.6. Confocal Microscopy and Image Analysis

**Figure 6:**
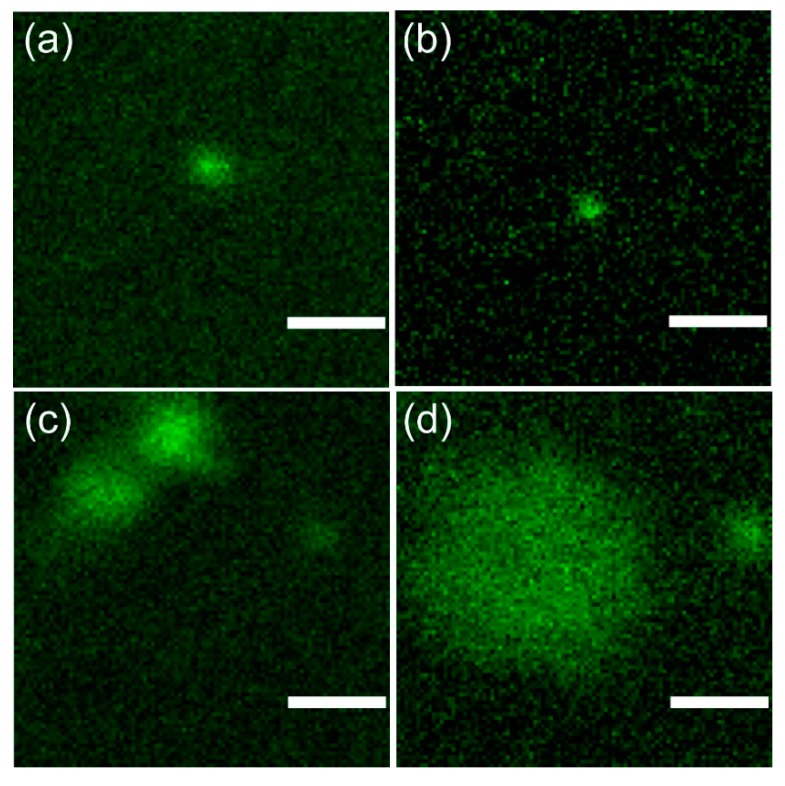
Representative fluorescence confocal micrographs of NP. For all images, the same field of view and pixel size was adopted, to allow a direct visual comparison of NP size. (**a**) SB-Ch NP (sol state), (**b**) SB-Ch NP (gel state), (**c**) QA-Ch NP (gel state), and (**d**) QA-Ch-*S*-pro NP (gel state). Scale bar: 1 µm.

Particle fluorescence allowed their visualization in both the sol and gel states by means of fluorescence confocal microscopy [13]. From particle images, their size could be inferred (Table 4, Figure 6) affording interesting information on the role of gelification on their aggregation status. Gelation did not affect the size of SB-Ch NP (Table 4, entries 1,2; Figure 6a,b) which remained close to the optical diffraction limit of the microscopy apparatus (~0.2 µm). Conversely, QA-Ch NP in the gel phase showed the presence of two main particle populations: one, with a size comparable to that of SB-Ch NP, the other twice as large (Table 4, entry 3; Figure 6c). A bimodal pattern was observed also with QA-Ch-*S*-pro NP, although in this case the smaller peak was about 0.6 µm and the larger was, on the average, more than 4 µm with a rather large dispersion as indicated by the relevant standard error (SE) values (Table 4, entry 4; Figure 6d). These findings strongly suggest a moderate aggregation of QA-Ch NP, each aggregate possibly consisting of two particles sticking together, in the sol state, and a much larger aggregate state for QA-Ch-*S*-pro NP in the gel state. These data encouraged us to continue the study only with NP based on QA-Ch and SB-Ch.

### 3.7. Micro-Rheological Characterization of NP Mucoadhesive Properties

**Figure 7:**
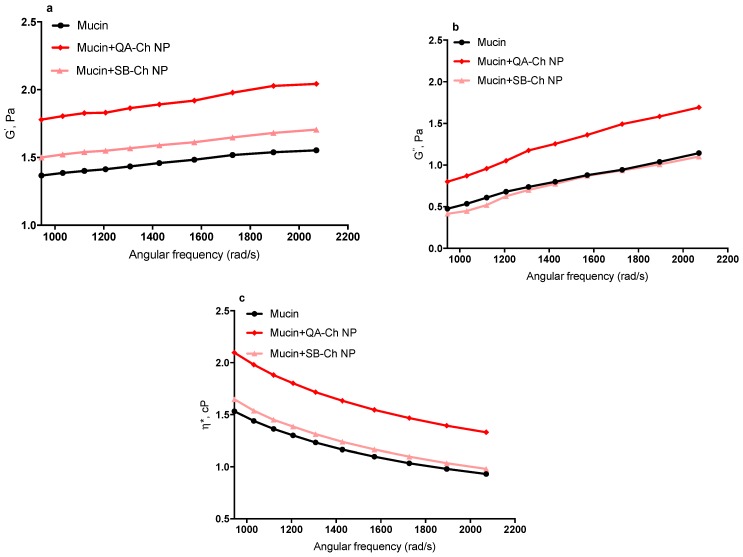
Elastic modulus, G’ (**a**), viscous modulus, G’’ (**b**), and complex viscosity, η* (**c**), of QA-Ch and SB-Ch NP with respect to mucin dispersion.

The elastic (or storage) modulus, G’, the viscous (or loss) modulus, G’’, and the complex viscosity, η*, are reported in Figure 7. As can be seen, there was an increase in η* of mucin dispersion in the presence of NP based on either QA-Ch or SB-Ch, compared to plain mucin dispersion. The increase of η* was reflected in the increase of both G’ and G’’ moduli. In particular, the increase in G’ is indicative of the development of an inter-connected microstructure between mucin macromolecules and NP based on QA-Ch, resulting in a stronger mucoadhesivity of these NP with respect to the NP based on SB-Ch. However, G’ for NP based on SB-Ch was higher than G’ for mucin, due to the intrinsic mucoadhesivity of Ch, i.e., the pristine material used to prepare SB-Ch. These data demonstrate that the sign of the NP surface charge can actually influence their mucoadhesivity. Moreover, it is known that the mucus glycoproteins bear negatively charged sialic moieties that are capable of forming ionic bonds with oppositely charged chemical species [31].

### 3.8. In Vivo Tests

**Figure 8:**
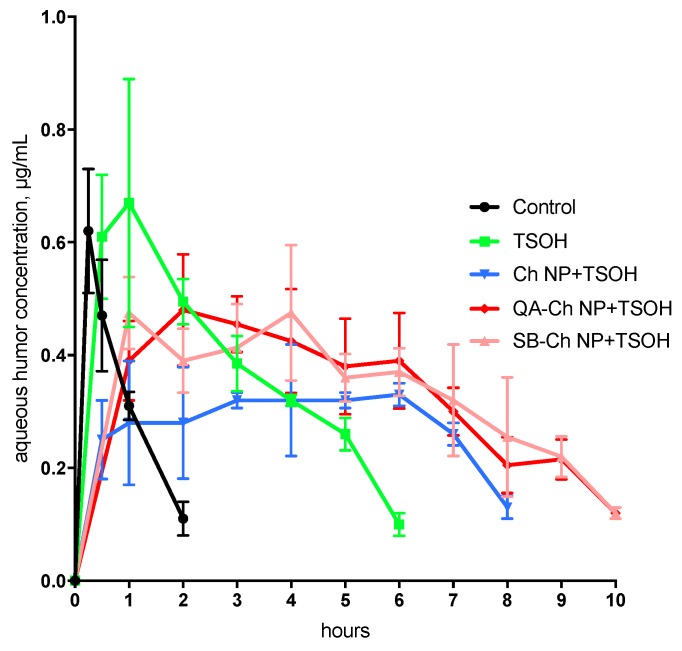
Pharmacokinetics in the aqueous following instillation of ophthalmic drops. Means ± SD (*n* = 6). Control, TSOH, and Ch NP+TSOH profiles are taken from Fabiano et al. [13].

During each experiment, we observed that all the ophthalmic drops instilled in rabbit eyes caused no conjunctival/corneal edema and/or hyperemia. The pharmacokinetic profiles in the aqueous and the relative AUC values are reported in Figure 8 and Table 5, respectively. The results of the in vivo tests shown in Figure 8 demonstrate the ability of the TSOH containing NP based on QA-Ch or SB-Ch to increase the 5-FU bioavailability with respect to the control, TSOH, and Ch NP-containing TSOH [13]. Indeed, the AUC values relative to QA-Ch NP+TSOH and SB-Ch NP+TSOH listed in Table 5, are significantly higher than those relative to the control, TSOH, and Ch NP+TSOH, with a concentration plateau in the range 1–10 h. These results demonstrate that the QA-Ch NP+TSOH and SB-Ch NP+TSOH systems have much more ability to prolong the drug precorneal residence time than the control, TSOH, or Ch NP-containing TSOH. In view of the ability of NP to retain the drug for longer, the plateau in the 1–10 h range is in-keeping with the hypothesis of an intraocular drug absorption controlled by gel erosion in pre-corneal area accompanied by release of drug-loaded NP that are then internalized in corneal cells.

It is interesting to note that no differences were seen, in Figure 8, in the concentration in the aqueous vs. time profiles between any of the three NP-containing TSOH formulations. This can be ascribed to the presence in all formulations of a significant 5-FU dose fraction not entrapped in NP, but free to permeate across the cornea by passive diffusion. Such a drug fraction is unaffected by the different NP ability to be internalized in corneal cells, which can indeed be influenced by the NP surface characteristics.

The data altogether demonstrate the importance of NP mucoadhesion properties and of their ability to interact with the vehicle. In fact, QA-Ch NP-containing TSOH showed an AUC value higher than that for TSOH or Ch NP+ TSOH probably thanks to their fixed positive charges that prolong the drug retention time and increase the drug contact with the anterior surface of the eyes and thereby, enhance ocular absorption via paracellular transport through the tight junctions of corneal epithelia [16]. On the other hand, SB-Ch NP+TSOH showed an AUC value similar to that of QA-Ch NP+TSOH probably due to its ability to slow down the 5-FU release from the vehicle, as demonstrated in Section 3.5.

These present results are in agreement with those shown in a preceding paper where it was found that the more effective NP were able to concurrently adhere to the ocular surface and strongly interact with the drug molecules in solution [10]. After all, although less mucoadhesive than QA-Ch NP, the SB-Ch NP also showed some mucoadhesivity due to the intrinsic mucoadhesivity of the Ch backbone.

## 4. Conclusions

These results indicate that the present SB-Ch NP-containing thermosensitive hydrogels are able to prolong 5-FU ocular residence thanks to the synergistic effect of negative charges on NP surface and positive charges present in the TSOH. Furthermore, in the case of QA-Ch NP-containing thermosensitive hydrogels the presence of positive charges on NP surface prolongs their contact with corneal and conjunctival surfaces that are negatively charged. As a result, both NP-containing QA-Ch-based and SB-Ch-based TSOH were able to increase the ocular 5-FU bioavailability. NP-containing thermosensitive hydrogels could be administered as conventional eyedrops and still represent an alternative, more effective formulation than the commercial 5-FU eyedrops, with reduced 5-FU applied dose and instillation frequency. However stability studies of the formulations must be carried out in the future to understand if they can really be commercialized.

## Figures and Tables

**Table 1:** Characteristics of medicated nanoparticles (NP) determined immediately after their preparation.

NP Type	Nanoparticle Size, nm (Polydispersity Index)	ζ, mV	EE%
NP quaternary ammonium-Ch conjugate (QA-Ch)	294.3 ± 60.6 (0.4 ± 0.2)	+9.1 ± 0.4	18.4 ± 4.0
NP QA-Ch-*S*-pro	340.7 ± 100.1 (0.3 ± 0.1)	+9.5 ± 0.1	18.3 ± 0.2
NP SB-Ch	390.6 ± 50.3 (0.2 ± 0.1)	−3.5 ± 0.1	15.6 ± 1.0

**Table 2:** Results of dynamic dialysis data plotted in Figure 3 and obtained according to Equation (2).

Formulation	Modulus of Straight-Line Slope ± SD (h^−1^)	*r* ^2^	Interaction ^a^ %
Control	0.44 ± 0.04	0.97	-
QA-Ch	0.23 ± 0.03	0.94	47.7
NP QA-Ch	0.37 ± 0.03	0.98	15.9
QA-Ch-*S*-pro	0.28 ± 0.04	0.93	36.4
NP QA-Ch-*S*-pro	0.37 ± 0.03	0.98	15.9
SB-Ch	0.29 ± 0.06	0.90	34.1
NP SB-Ch	0.41 ± 0.06	0.93	6.8

^a^ Bound fraction.

**Table 3:** Characteristics of 5-FU release from NP-containing TSOH.

Formulation	Slope of √t Plot (%/√h)	Cumulative Release at 5 h (%)
Ch NP+TSOH ^a^	3.34 ± 0.32	59.50 ± 0.90
QA-Ch NP+TSOH	3.14 ± 0.39	52.23 ± 1.07
QA-Ch-*S*-pro NP+TSOH	3.64 ± 3.12	65.93 ± 5.70
SB-Ch NP+TSOH	2.43 ± 0.21 *	37.00 ± 3.82 *

^a^ Data taken from Fabiano et al., 2017 [13]. * Significantly different from all the others (*p* < 0.05).

**Table 4:** Size of the particles as determined by confocal fluorescence microscopy. Each entry refers to one experiment where the size was calculated as the average of five full width at half maximum (FWHM) measurements (see Materials and Methods section).

Entry	Particle (Status)	Mean Size ± SE (µm)
1	SB-Ch NP (sol)	0.27 ± 0.02
2	SB-Ch NP (gel)	0.33 ± 0.02
3	QA-Ch NP (gel)	0.70 ± 0.07; 0.29 ± 0.01
4	QA-Ch-*S*-pro NP (gel)	4.26 ± 1.67; 0.61 ± 0.15

**Table 5:** AUC data obtained in vivo in rabbit eyes.

Formulation	AUC_0–10_, μg h/mL	AUC_rel_
Control ^a^	0.62 ± 0.1	-
TSOH ^a^	2.32 ± 0.26 *	3.7
Ch NP+TSOH ^a^	2.23 ± 0.39 *	3.6
QA-Ch NP+TSOH	3.30 ± 0.52 *^,^**	5.3
SB-Ch NP+TSOH	3.34 ± 0.53 *^,^**	5.4

^a^ Data taken from Fabiano et al., 2017 [13]; * *p* < 0.05 versus control, ** *p* < 0.05 versus TSOH, and Ch NP+TSOH.
